# Preferential Effects of Cariprazine on Counteracting the Disruption of Social Interaction and Decrease in Extracellular Dopamine Levels Induced by the Dopamine D_3_ Receptor Agonist, PD-128907 in Rats: Implications for the Treatment of Negative and Depressive Symptoms of Psychiatric Disorders

**DOI:** 10.3389/fpsyt.2021.801641

**Published:** 2022-01-12

**Authors:** Jan Kehr, Fu-Hua Wang, Fumio Ichinose, Shimako Yoshitake, Bence Farkas, Béla Kiss, Nika Adham

**Affiliations:** ^1^Pronexus Analytical AB, Bromma, Sweden; ^2^Department of Physiology and Pharmacology, Karolinska Institutet, Stockholm, Sweden; ^3^Pharmacological and Drug Safety Research, Gedeon Richter Plc., Budapest, Hungary; ^4^Allergan plc, Madison, NJ, United States

**Keywords:** cariprazine, schizophrenia, dopamine D_3_ receptor, huddling, microdialysis

## Abstract

The negative and cognitive symptoms of schizophrenia and related disorders may be due to reduced dopaminergic tone in cortical brain areas. Alteration in the function of dopamine (DA) D_3_ receptors may play a role in this cortical hypofunctionality and underlie the deficits in social behaviors and cognitive functions in schizophrenia. Cariprazine is a potent DA D_3_-preferring D_3_/D_2_ receptor partial agonist that is approved for the treatment of schizophrenia and bipolar disorder. The objective of the study was to compare the abilities of cariprazine, aripiprazole (another DA receptor partial agonist with more D_2_ receptor preference), and ABT-925 (a selective DA D_3_ antagonist) to counteract the social deficit and neurochemical alterations induced by the D_3_ receptor-preferring agonist (+)-PD 128907 (PD) in rats. Administration of PD (0.16 mg/kg; s.c.) induced a marked (−72%) but short-lasting disruption of the defensive social aggregation behavior (huddling) in the first 10-min period. Cariprazine at all doses (0.1, 0.3, 1 mg/kg; p.o.) almost completely abolished the PD-induced disruption of huddling. Likewise, ABT-925 (3 mg/kg; p.o.) and to a lesser extent aripiprazole (20 mg/kg; p.o.) were effective in blocking the PD-induced disruption of huddling. As measured by microdialysis, the highest dose of cariprazine prevented a PD-induced decrease in DA levels (40–80 min post PD dose) in the medial prefrontal cortex (mPFC), whereas aripiprazole did not have a significant effect. ABT-925 significantly counteracted the effect of PD at 80 min post-dose. In the nucleus accumbens (nAcc) shell, the highest dose of cariprazine, as well as ABT-925 and aripiprazole, significantly reversed the PD-induced decrease in DA levels. Taken together, these data provide behavioral and *in vivo* neurochemical evidence for the preferential DA D_3_ receptor action of cariprazine in the rat. This property of cariprazine may offer therapeutic benefits against the cognitive deficits and negative/depressive symptoms of schizophrenia and related disorders.

## Introduction

Cariprazine (Vraylar^®^ in USA; Reagila^®^ in Europe) is approved in the USA for the acute and maintenance treatment of schizophrenia, as well as the acute treatment of manic or mixed episodes associated with bipolar I disorder and bipolar depression in adults. It is also approved by the EMA for the treatment of schizophrenia in adults and is in clinical development as an adjunctive treatment for major depressive disorder. Cariprazine acts as a potent dopamine (DA) D_3_ receptor-preferring D_3_/D_2_ receptor partial agonist, as well as a partial agonist of serotonin 5-HT_1A_ receptors (1). Cariprazine can be distinguished from currently used atypical antipsychotics by its higher *in vitro* binding affinity (K_i_) and selectivity for human D_3_ receptors (0.085 nM) compared to D_2L_ (0.49 nM) and D_2S_ (0.69 nM) receptors (1, 2). In addition, cariprazine displays subnanomolar affinity for serotonin 5-HT_2B_ receptors; nanomolar affinity for serotonin 5-HT_1A_, 5-HT_2A_, and histamine H_1_ receptors; and low affinity for serotonin 5-HT_7_, 5-HT_2C_, and adrenergic alpha receptors (1).

The D_3_ receptor is thought to play a role in mood (3) and cognition (4). Cariprazine was developed based on the hypothesis that a compound with high affinity for D_3_ and D_2_ receptors may provide benefits for treating the affective and cognitive deficits associated with schizophrenia and bipolar disorder (5, 6). *In vivo*, cariprazine achieves high occupancy of both D_3_ and D_2_ receptors at doses that produce antipsychotic-like effects in rats (7) and at clinically active dose ranges in patients with schizophrenia (8). Cariprazine's pharmacological profile differs from that of other atypical antipsychotics such as aripiprazole, clozapine, olanzapine, and risperidone, which have varying levels of *in vitro* affinity for D_3_ receptors but fail to show significant D_3_ receptor occupancy at doses that produce antipsychotic-like effects in rats (9) and/or at clinically relevant doses in patients with schizophrenia (10, 11). These data indicate that cariprazine can modulate the activity of D_3_ receptors *in vivo* to a greater extent than other atypical antipsychotics in clinical use.

In animal models of schizophrenia, cariprazine reversed PCP- or MK-801-induced behavioral effects such as hyperlocomotion (7), demonstrating putative efficacy for treating the positive symptoms of schizophrenia. In a follow-up study in mice, cariprazine significantly diminished PCP-induced cognitive deficits in wild-type mice, but not in D_3_ receptor knockout mice (12). In addition, two recent studies provide further support for cariprazine's ability to ameliorate PCP-induced cognitive and social deficits in adult rats (13) and in a PCP-neurodevelopmental model of schizophrenia in rats (14). Together, these results from PCP models of schizophrenia suggest that cariprazine may exert beneficial effects on the cognitive and social/affective functions disrupted by PCP, at least in part through its high affinity for and occupancy of D_3_ receptors.

Indeed, in addition to cariprazine's efficacy (vs. placebo) in patients with acute exacerbation of schizophrenia (15–17), cariprazine has also demonstrated enhanced efficacy (vs. risperidone) for treating the negative symptoms of schizophrenia in patients with predominantly negative symptoms (18, 19). These data suggest that cariprazine displays a differentiated clinical profile compared to other atypical antipsychotic medications, which may be driven by its unique D_3_ receptor mechanism.

It has been demonstrated that dopamine D_3_ receptor-preferring agonists such as 7-OH-DPAT (20), (+)-PD 129807 (PD) (21, 22), and pramipexole (23) cause biphasic behavioral changes in rats: at low doses they cause yawning, whereas at high doses they cause increased penile grooming, sniffing, hypothermia, locomotor stimulation, and stereotypy (22–27). It has been proposed that induction of yawning elicited by these agonists at low doses is mediated through the activation of dopamine D_3_ receptors (22, 26, 27). At low doses, similar to those that induce yawning, (±)-7-OH-DPAT and (+)-PD 128907 also caused a dose-dependent disruption of huddling, a normal social behavior involving direct body contact in rats. Thus, the alteration of huddling elicited by low, D_3_ receptor-selective doses of (+)-PD 128907 and (±)-7-OH-DPAT are considered a useful behavioral model for dopamine D_3_ receptors (28–30). (+)-PD 128907-induced disruption of huddling can be reversed by selective dopamine D_3_ receptor antagonists such as A-437203 (ABT-925) or A-690304 (30, 31) or partially reversed by antipsychotics (29).

The objective of this study was to evaluate the ability of acute cariprazine administration to counteract the disruptive effect of the D_3_ receptor-preferring dopamine agonist, (+)-PD 128907, on huddling behavior in rats compared to the D_2_/D_3_ receptor partial agonist antipsychotic aripiprazole (32) and the selective D_3_ receptor antagonist ABT-925 (31). This study also aimed to determine the role of D_3_ receptors in dopaminergic neurotransmission. To this end, we used dual-probe microdialysis in the prefrontal cortex (mPFC) and nucleus accumbens (nAcc) shell of awake rats to measure the extracellular levels of DA and its metabolites, dihydroxy-phenylacetic acid (DOPAC) and homovanillic acid (HVA), in response to cariprazine, ABT-925, and aripiprazole before a (+)-PD 128907 challenge.

## Materials and Methods

### Animals

Male Sprague-Dawley rats (8–10 weeks of age, weighing 300–350 g on the day of the experiment) were used in the study. The rats weighed 250–275 g upon reception from Janvier Labs, France, and were allowed a minimum acclimatization period of 1 week prior to any experiments. No prophylactic or therapeutic treatments were administered during the acclimatization period. Animals were kept in a controlled environment (22 ± 1°C; 45–50% rel. humidity) on a 12 h dark/12 h light cycle (40 Lux, lights on at 6:00 AM). The rats had free access to standard laboratory chow (RM1A(P), SDS, Scanbur, Sweden) and tap water until the time of the experiments. The rats were housed in groups of five in Eurostandard type IV cages (595 × 380 × 200 mm, LWH, floor area 1,820 cm^2^) with wire lids (Tecniplast, Buguggiate, Varese, Italy) and aspen bedding (Tapvei, Estonia). Aspen gnawing bricks, aspen arcades, or tunnels (Tapvei) were placed in each cage as environmental enrichment. All rats were examined and weighed prior to study initiation to assure adequate health and suitability. Rats were randomly assigned to treatment groups.

### Test Compounds

Cariprazine hydrochloride salt, aripiprazole free base, and ABT-925 were provided by Allergan, NJ, USA. (+)-PD 128907 hydrochloride was purchased from Tocris Bioscience, UK. All other chemicals were purchased from Sigma-Aldrich (St. Louis, MO, USA).

### Groups and Doses

#### Huddling Study

The huddling study included 10 groups of eight rats each. The test compounds cariprazine, aripiprazole, or ABT-925 (3 ml/kg total volume) were administered orally (p.o.) 30 min prior to administration of either (+)-PD 128907 or saline. (+)-PD 128907 or saline (1 ml/kg total volume) were administered subcutaneously (s.c.). The vehicle for the p.o. administrations consisted of 0.4% (v/v) acetic acid in saline (20 μl acetic acid in 5 ml saline). The vehicle for the s.c. injections was saline. Fresh formulations of the test compounds were prepared on the day of each experiment. The doses and treatments for each group are summarized in Table 1 below.

**Table 1:** The doses and the order of the test compounds for the huddling study.

**Group**	**Treatment 1 (*t* = −30 min)**	**Treatment 2 (*t* = 0 min)**
1	Vehicle p.o.	Vehicle s.c.
2	Vehicle p.o.	(+)-PD 128907 (0.16 mg/kg s.c.)
3	ABT-925 (3 mg/kg p.o.)	Vehicle s.c.
4	Cariprazine (1.0 mg/kg p.o.)	Vehicle s.c.
5	Aripiprazole (20 mg/kg p.o.)	Vehicle s.c.
6	ABT-925 (3 mg/kg p.o.)	(+)-PD 128907 (0.16 mg/kg s.c.)
7	Cariprazine (0.3 mg/kg p.o.)	(+)-PD 128907 (0.16 mg/kg s.c.)
8	Cariprazine (0.1 mg/kg p.o.)	(+)-PD 128907 (0.16 mg/kg s.c.)
9	Cariprazine (1.0 mg/kg p.o.)	(+)-PD 128907 (0.16 mg/kg s.c.)
10	Aripiprazole (20 mg/kg p.o.)	(+)-PD 128907 (0.16 mg/kg s.c.)

*The doses refer to free bases*.

#### Microdialysis Study

The microdialysis study included six groups of seven rats each. The test compounds cariprazine (0.1, 0.3, and 1 mg/kg), aripiprazole (20 mg/kg), or ABT-925 (3 mg/kg) were administered p.o. at the same doses and volumes as in the huddling study, 20 min prior to administration of (+)-PD 128907 (0.16 mg/kg) or saline (s.c.). Fresh formulations of the test compounds were prepared on the day of each experiment.

### Experimental Procedures

#### Social Behavior (Huddling)

The protocol for the huddling study was modified from that described by Kagaya and colleagues (28). Examination of huddling behavior can be automated (by video recording) and then visually evaluated afterwards. Four rats (one from each housing cage) were randomly selected and placed in the housing room. Two rats were randomly selected to be scored for huddling behavior and distinctly marked with a non-toxic permanent marker. All four rats were then placed in a new cage (2154F; floor area 940 cm^2^, height 21 mm; Tecniplast, Italy) with aspen bedding and left to habituate for 24 h. Water and food pellets were available *ad libitum*. On the day of the experiment, the cage was transferred to the examination room for video recordings. Following 10 min of acclimatization in the examination room, the marked rats were administered the vehicle or the test substance (p.o.) and placed back into the home cage. After 30 min, the two marked rats were administered (+)-PD 128907 (0.16 mg/kg) or vehicle (s.c.) and placed back into the cage with the remaining two naïve rats. The motor behavior of all rats was recorded on video for 90 min using the Smart 3.0 Video Tracking System (Panlab, Harvard Apparatus, USA). Huddling was defined as the total time each of the marked rats spent in direct body contact with a group of two or three other rats. The video recordings were examined and the total huddling time for each marked rat was determined by a blinded experimenter.

#### Surgical Procedure

**Figure 1:**
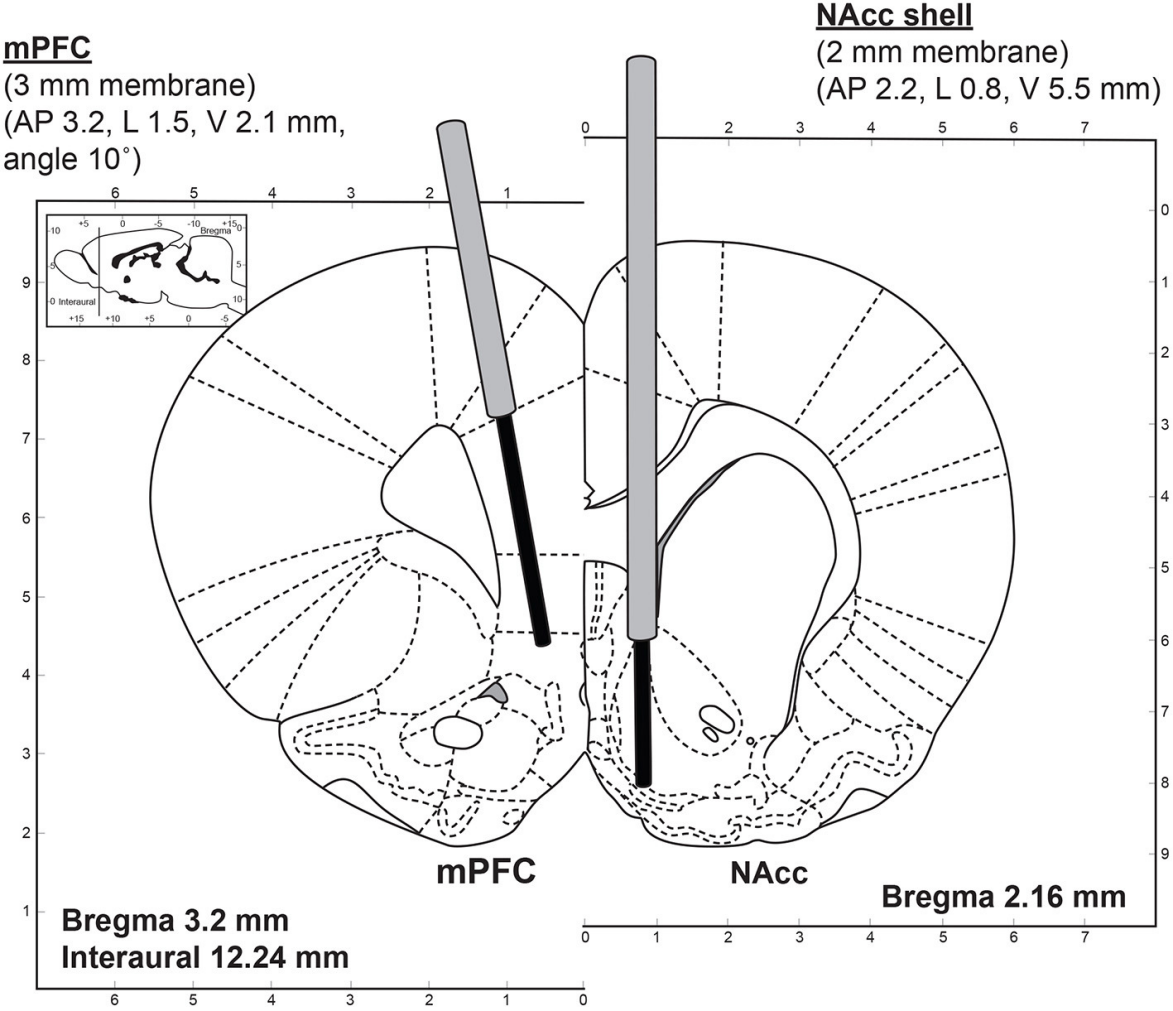
Dual-probe microdialysis—an illustration of the stereotaxic placement of the microdialysis probes into the mPFC (3 mm membrane length) and nAcc shell (2 mm membrane length) at coordinates determined using the rat brain atlas (35).

The microdialysis experiments were carried out in awake rats following a previously described protocol (33, 34). The initial stereotaxic surgery was performed under aseptic conditions. The rats were anesthetized with isoflurane using a Univentor 400 anesthesia unit (AgnThos, Lidingö, Sweden) and placed in a stereotaxic frame (David Kopf Instruments, Tujunga, CA, USA) in a flat skull position with the incisor bar set to −3.2 mm. After the induction of anesthesia but before the surgery, each animal received 5 mg/kg (s.c.) carprofen (“Rimadyl”, Pfizer). An ocular lubricant gel (Viscotears, Novartis) was applied to both eyes to prevent drying of the cornea during the surgical procedure. During the operation, the body temperature of the animal was controlled using a thermometer and a heating pad maintained at 37°C by a CMA/105 temperature controller (CMA/Microdialysis, Stockholm, Sweden). The site of the surgical incision was clipped of hair, disinfected with chlorhexidine solution (1%), and injected s.c. with the local anesthetic Marcain (bupivacaine). A midline scalp incision 1.5–2 cm in length was made and the incision was kept open using homeostatic forceps. After exposing the skull, two small holes were drilled on each side of the brain for the implantation of the guide cannulae using a fine trephine drill. Two more holes were drilled for the anchor screws. Two micro screws were then placed into the skull. One guide cannula (Eicom Corp., Kyoto, Japan) was implanted into the mPFC at the following coordinates: AP +3.2 mm; L −1.5 mm; DV −2.1 mm, at a 10° angle, such that the final DV coordinate was−5.0 mm for the tip of the microdialysis probe. The second guide cannula was implanted into the nAcc shell in the contralateral hemisphere: AP +2.2 mm; L +0.8 mm; DV −5.5 mm, such that the final DV coordinate was −7.5 mm for the tip of the microdialysis probe. This arrangement allowed both brain structures to be targeted in the same rat. The coordinates were made in reference to bregma and the dural brain surface and were determined using the Paxinos and Watson stereotaxic atlas (35). The final placement of the probes is shown in Figure 1.

The guide cannulae were fixed firmly to the skull surface using dental cement (Dentalon Plus, Heraeus, Germany). After completing the surgery, the animals were closely supervised and allowed to recover over the following 7 days. After surgery, the rats were housed individually in their home cages (Eurostandard III H, Tecniplast, Italy) until the day of the microdialysis experiment. During this period, the general health status of the animals was monitored regularly.

### Microdialysis Sampling

On the day of the microdialysis experiment, the microdialysis probes (Eicom A-I: 0.22 mm o.d., 50 kDa cut-off, mPFC: 3 mm membrane length, nAcc: 2 mm membrane length) were inserted into each respective guide cannula of the awake rat. The probes were perfused with artificial cerebrospinal fluid (aCSF) solution (148 mM NaCl, 4 mM KCl, 0.8 mM MgCl_2_, 1.4 CaCl_2_, 1.2 mM Na_2_HPO_4_, 0.3 mM NaH_2_PO_4_, pH 7.2) at a constant flow-rate of 1 μl/min. The rat was placed into a microdialysis system (CMA/Microdialysis, Stockholm, Sweden) equipped with a 2-channel swivel (TCS2-23; ALS, Tokyo, Japan), which allowed the rat to move freely within its home cage during microdialysis. The rat was allowed to habituate to the new environment for 120–150 min. Following this stabilization period, the microdialysis samples were collected in 20-min intervals. The first 3 samples were collected to determine the basal extracellular levels of neurotransmitters and their metabolites. The test compound or vehicle was then administered, followed 20 min later by a s.c. injection of (+)-PD 128907. Samples were then collected for an additional 3 h. All microdialysis experiments were performed between 9 AM and 6 PM. After the experiment, the animals were sacrificed by isoflurane overdose and dislocation of the neck. Terminal blood was collected intracardially. The brains were rapidly removed, frozen on dry ice, and stored at −80°C for additional analysis of tissue biomarkers or histological verification of the microdialysis probe placement.

### Locomotor Activity Test

The locomotor activity of rats undergoing microdialysis sampling was monitored using a single-beam activity frame (44 x 30 cm; ACTIMO 10, Shintechno, Japan) placed around the lower part of the Macrolon III cage in order to control for the effects of stress induced by handling and drug administration. This arrangement allowed for simultaneous locomotor activity recording and microdialysis sampling. The data were collected by counting and summarizing the overall activity (number of beam crossings) in 5-min intervals, which if necessary were further pooled into 20-min bins to match the frequency of microdialysis sampling.

### HPLC Analysis

The monoamines DA and 5-HT were measured by ion-exchange narrow bore column liquid chromatography with electrochemical detection as described previously (34). The HPLC system (HTEC-500, Eicom, Japan) included a pulse-free microflow pump, a degasser, and an amperometric detector equipped with a graphite electrode operating at +0.45 V vs. an Ag/AgCl reference electrode. Samples were injected using a CMA/200 Refrigerated Microsampler (CMA/Microdialysis) and the chromatograms were recorded and integrated using a computerized data acquisition system (DataApex, Prague, Czech Republic). DA and 5-HT were separated using a 200 x 2.0 I.D. mm column (CAX, Eicom, Japan). The mobile phase consisted of 0.1 M phosphate buffer at pH 6.0, 30 mM potassium chloride, and 28% (v/v) methanol. The detection limit (signal-to-noise ratio = 3) for DA and 5-HT was 0.5 fmol per 10 μl injected onto the column. The concentration of the acidic metabolites, DOPAC and HVA, in 3–5 μl microdialysis samples was determined using a second HPLC system with electrochemical detection (33, 34). Briefly, the HPLC system (HTEC-500, Eicom Corp., Kyoto, Japan) included a pulse-free microflow pump, a degasser, and an amperometric detector equipped with a glassy-carbon electrode operating at +0.45 V vs. an Ag/AgCl reference electrode. Samples were injected using a CMA/200 Refrigerated Microsampler (CMA/Microdialysis). The chromatograms were recorded and integrated using a computerized data acquisition system (DataApex, Prague, Czech Republic). DOPAC and HVA were separated using a 150 x 2.1 I.D. mm column (CA5-ODS, Eicom Corp., Kyoto, Japan). The mobile phase consisted of 0.1 M phosphate buffer at pH 6.0, 0.13 mM EDTA, 2.3 mM sodium-1-octanesulfonate, and 20% (v/v) methanol.

### Data Presentation and Analysis

Raw data were entered into data files using a standard spreadsheet program (Microsoft Excel) and statistical analysis was performed using Prism 9 statistical software (GraphPad Software, USA) and differences are considered to be statistically significant at the *P* < 0.05 level. Values for figures showing the time courses of behavioral and microdialysis variables are presented as mean ± standard error of mean (SEM). The huddling behavior was counted in seconds and summarized in 10-min bins during 90 min post-treatment with (+)-PD 128907. The interaction of time and treatment between the (+)-PD 128907-treated group and the drug-treated groups were compared by the two-way RP ANOVA followed by Bonferroni's multiple comparison test. Differences between the saline and drug-treated groups, as well as the (+)-PD 128907-treated group and the (+)-PD 128907+drug-treated groups in 10 and 20 min bins, respectively, were analyzed by one-way ANOVA followed by Šidák's multiple comparison test. The figures are presented as box-and-whiskers including the median values, 25 and 75% values (boxes), and minimum and maximum values (whiskers). The normal distribution of data was evaluated by D'Agostino & Pearson test.

For graphic presentation of the microdialysis data, concentrations of monoamines and metabolites over time were expressed as the percentage of the basal concentrations at time 0 min. The mean (±SEM) basal levels were calculated from the three samples collected before drug treatment. The basal levels were compared using a Kruskal-Wallis test followed by Dunn's multiple comparison test. Differences between the treatments and the interaction of time and treatment were analyzed by repeated measures two-way ANOVA followed by Bonferroni's post-test and using the Geisser-Greenhouse correction for non sphericity of variables. The overall effects of the treatments were expressed as the differences in relative AUC_(0−180min)_ for each treated group compared to the theoretical 100% control values. The differences between the relative AUC_(0−180*min*)_ for the groups were analyzed by Kruskal-Wallis followed by Dunn's multiple comparison test. The figures are presented as box-and-whiskers with median, 25 and 75% and minimum-maximum values.

## Results

### Huddling Behavior

**Figure 2:**
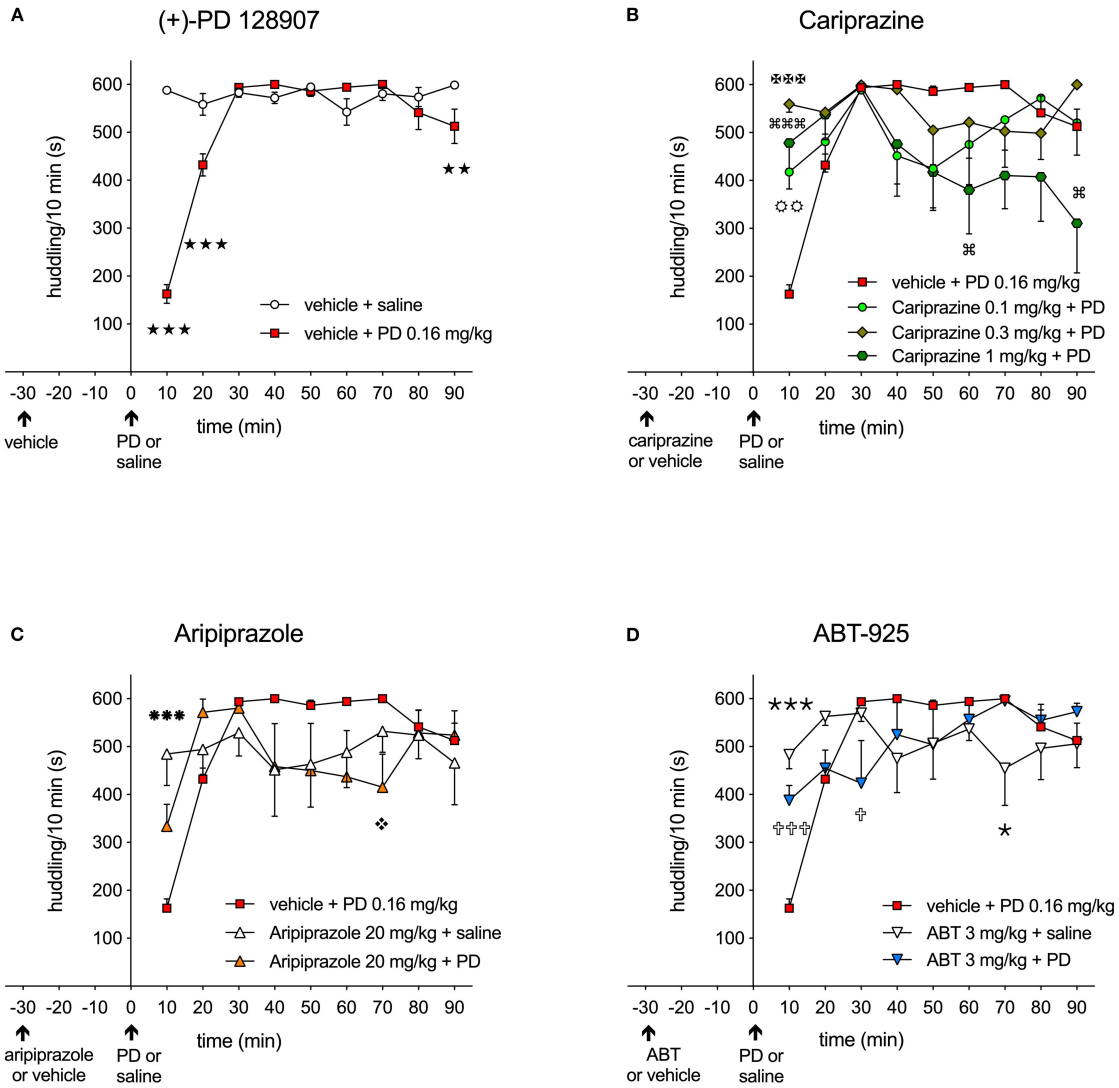
**(A)** Effect of the D_3_/D_2_ receptor agonist (+)-PD 128907 (0.16 mg/kg s.c.) on disruption of huddling behavior in rats over 10-minute intervals compared to the saline-injected group (stars); (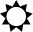

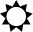

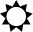
) *P* < 0.001, (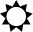

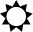
) *P* < 0.01; **(B)** The effect of cariprazine on attenuating the disruption of huddling induced by (+)-PD 128907; (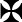

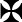
) *P* < 0.01 for the 0.1 mg/kg dose (circles), (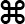

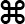

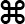
) *P* < 0.001 for the 0.3 mg/kg dose (Maltese crosses), (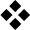

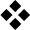

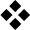
) *P* < 0.001, (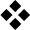) *P* < 0.05 for the 1 mg/kg dose (square loops); **(C)** The effect of aripiprazole (20 mg/kg, p.o.) on attenuating the disrupted huddling induced by (+)-PD 128907; (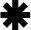

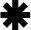

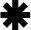
) *P* < 0.001 for aripiprazole + saline (snowflakes) vs. PD, (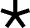) *P* < 0.05 for aripiprazole + PD (diamonds); **(D)** The effect of ABT-925 (3 mg/kg p.o.) on attenuating the disrupted huddling induced by (+)-PD 128907; (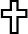

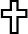

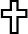
) *P* < 0.001, (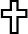) *P* < 0.05 for ABT + saline (thin stars) vs. PD; (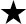

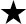

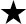
) *P* < 0.001,(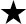) *P* < 0.05 for ABT + PD (crosses); mean ± SEM, *n* = 8; two-way repeated measures ANOVA followed by Bonferroni's multiple comparison test. ABT, ABT-925; PD, (+)-PD 128907.

The effect over time of the DA D_3_ receptor-preferring agonist (+)-PD 128907 (0.16 mg/kg, s.c.) on the disruption of huddling behavior of two drug-treated rats, compared to two naïve rats habituated in the same cage, is shown in Figure 2.

**Figure 3:**
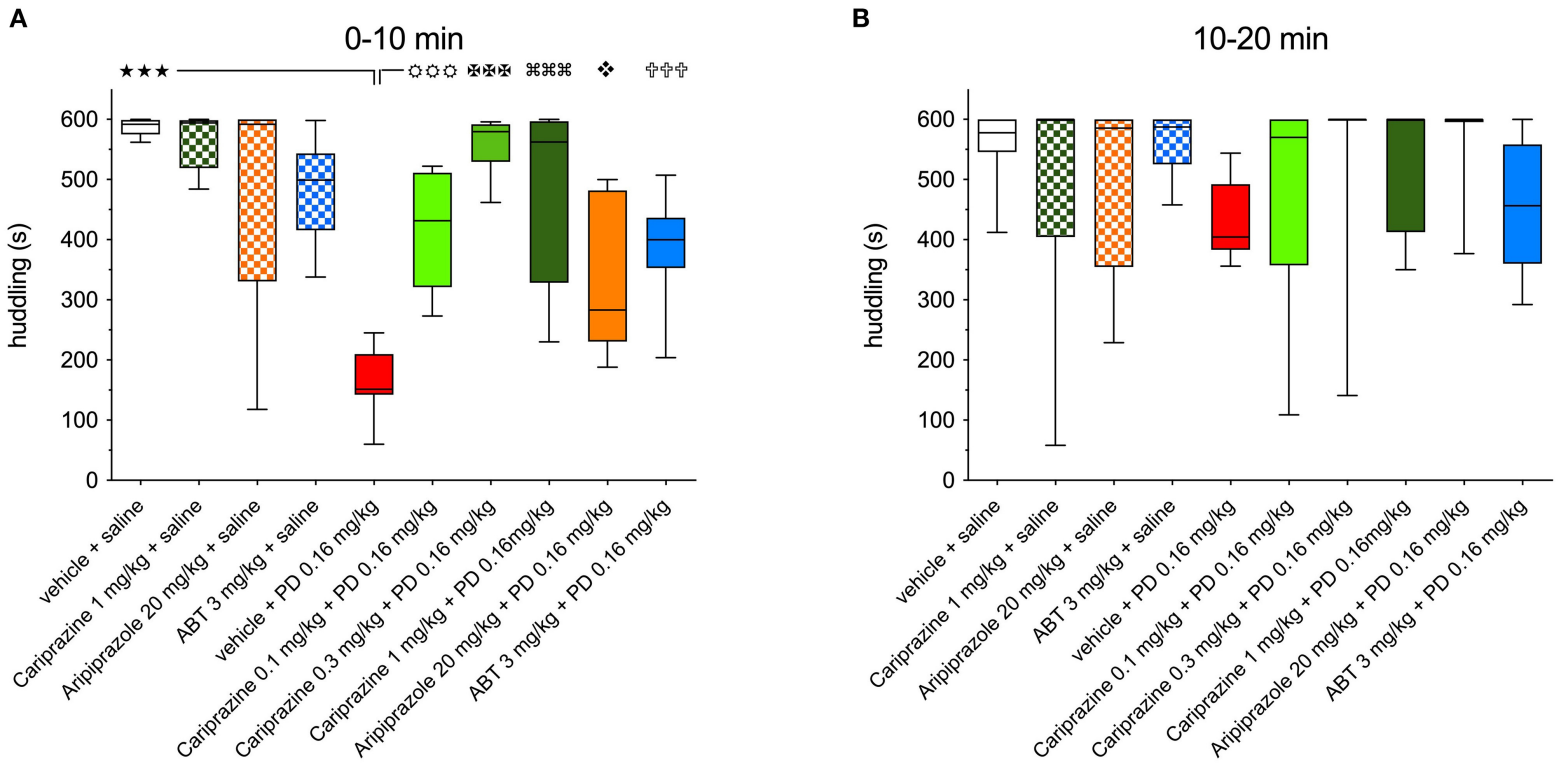
The effects of cariprazine, aripiprazole and ABT-925 on the (+)-PD 128907-induced disrupted huddling during **(A)** the first 0-10-minute period and **(B)** the second 10–20 min period post-treatment; mean ± SEM, *n* = 8; (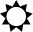

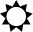

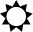
) *P* < 0.001; (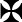

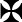

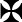
) *P* < 0.001, (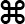

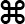

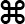
) *P* < 0.001, (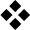

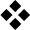

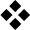
) *P* < 0.001 (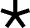) *P* < 0.05, (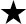

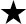

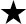
) *P* < 0.001, one-way ANOVA followed by Šidak's multiple comparison test.

The control, saline-treated rats showed a typical pattern of being tightly attached to each other in a clump, typically in one corner of the cage, during the entire recording period (600 s). Treatment with (+)-PD 128907 induced an immediate and significant (*P* < 0.001) reduction of huddling behavior during the first 10- and 20-min periods, as shown in Figure 2A. Thereafter, the treated rats returned to socializing with the naïve group. The time spent huddling between the groups was significantly different for both the treatment [F_(1, 14)_ = 28.98; *P* < 0.001] and the interaction of time and treatment [F_(8, 112)_ = 37.84; *P* < 0.001] as revealed by two-way repeated measures ANOVA followed by Bonferroni's post-test. Pre-treatment with cariprazine attenuated the disrupted huddling induced by (+)-PD 128907 during the first 10 min post-treatment, as shown in Figures 2B, 3A. The overall time spent huddling during the 90-min recording period between the groups was significantly different for the interaction of time and treatment [F_(24, 224)_ = 3.105; *P* < 0.001] but not for the treatment [F_(3, 28)_ = 1.99; P = 0.139]. However, for the first 10-min period, there was a marked and significant attenuation of disrupted huddling for both the higher doses of cariprazine (*P* < 0.001) as well as the lowest tested dose (*P* < 0.01). In addition, the highest dose of cariprazine tended to disrupt huddling at the end of the recording period (between 60 and 90 min) compared to the group treated with (+)-PD 128907 alone (Figure 2B).

The effects of aripiprazole and the selective DA D_3_ receptor antagonist ABT-925 on disrupted huddling induced by (+)-PD 128907 are shown in Figures 2C,D, respectively. Aripiprazole tended to attenuate the effect of (+)-PD 128907 during the first 10-min period, but this effect was not statistically significant (Figure 2C). Notably, aripiprazole showed a tendency to disrupt the huddling at the end of the recording period at 70 min (*P* < 0.05) compared to the group treated with (+)-PD 128907 alone, similarly to the highest dose of cariprazine. The time spent huddling was significantly different for the interaction of time and treatment [F_(16, 168)_ = 4.391; *P* < 0.001] but not for treatment with aripiprazole [F_(2, 21)_ = 0.012; P = 0.834]. However, the effect of aripiprazole was statistically significant when comparing the mean levels of huddling at 10-min intervals for all tested compounds by one-way ANOVA followed by a Šidak's multiple comparison test (Figure 3A). ABT-925 significantly (*P* < 0.001) but not completely attenuated (+)-PD 128907-induced disruption of huddling during the first 10 min post-treatment (Figure 2D). The overall time spent huddling during the 90-min recording period between groups was significantly different for the interaction of time and treatment [F_(16, 168)_ = 4.488; *P* < 0.001] but not for the treatment [F_(2, 21)_ = 0.012; *P* = 0.988].

Comparisons of the ability of all three test compounds to attenuate the (+)-PD 128907-induced disruption of huddling during the first and second 10-min post-dose intervals are shown in Figures 3A,B, respectively.

(+)-PD 128907 induced a marked and significant (*P* < 0.001) disruption of huddling during the first 10-min period. This can be compared to pretreatment with cariprazine, ABT-925 or aripiprazole before s.c. saline, which did not significantly impact time spent huddling compared to the vehicle + saline group. However, pretreatment with cariprazine or ABT-925 before (+)-PD 128907 administration markedly (*P* < 0.001) attenuated the (+)-PD 128907-induced disruption of huddling. Aripiprazole produced a similar but slightly weaker (*P* < 0.01) effect. Finally, as already shown in Figure 2, (+)-PD 128907-induced disruption of huddling was short lasting, as the rats returned to their social behavior and formed a clump (huddle) during the second 10-min period (Figure 3B). ABT, ABT-925; PD, (+)-PD 128907.

### Dual-Probe Microdialysis

#### Basal Levels of DA, DOPAC and HVA in the Rat mPFC and nAcc Shell

The basal concentrations of DA and its metabolites were calculated from the mean values of three fractions collected from each individual animal during the pre-drug period (−60 to 0 min) and then calculated as a mean ± standard error of the mean (SEM) for each treated group, *n* = 7 rats. The basal extracellular levels of DA, DOPAC, and HVA (expressed in fmol/10 μl) in the microdialysates from vehicle and drug-treated rats are presented in Supplementary Table S1.

The mean basal extracellular levels of DA, DOPAC, and HVA of vehicle and drug-treated groups were compared using Kruskal-Wallis test followed by Dunn's multiple comparison test. There were no significant differences between the groups when each group was compared to all other groups.

#### Effects of Cariprazine, Aripiprazole, and ABT-925 on DA Levels in the Rat mPFC and nAcc Shell Following (+)-PD 128907 Challenge

**Figure 4:**
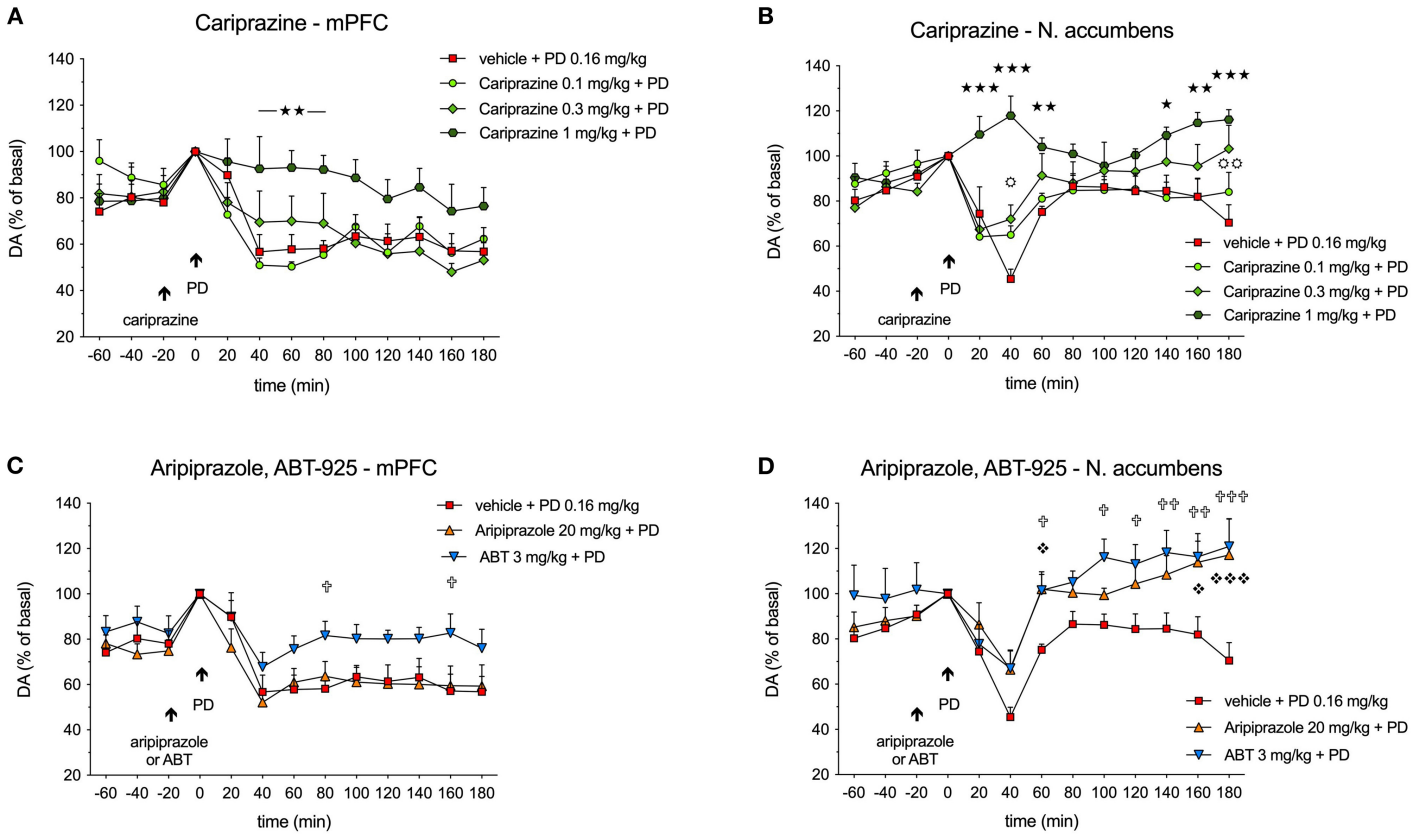
Effects of **(A,B)** cariprazine, and **(C,D)** aripiprazole and ABT-925 on (+)-PD 128907-induced decreases in extracellular DA levels in the mPFC and nAcc shell regions of awake rats; (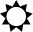

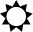

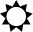
) *P* < 0.001, (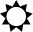

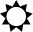
) *P* < 0.01, (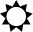) *P* < 0.05 for cariprazine (1 mg/kg) + PD vs. PD alone (stars); (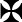

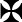
) *P* < 0.01, (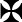) *P* < 0.05 for cariprazine (0.3 mg/kg) + PD vs. PD alone group (circles); (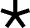

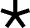

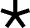
) *P* < 0.001, (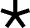) *P* < 0.05 for aripiprazole + PD vs. PD alone (diamonds); (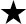

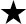

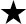
) *P* < 0.001, (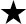

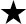
) *P* < 0.01, (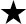) *P* < 0.05 for ABT + PD vs. PD alone (crosses); mean ± SEM, *n* = 7; two-way repeated measures ANOVA followed by Bonferroni's multiple comparison test. ABT, ABT-925; PD, (+)-PD 128907.

**Figure 5:**
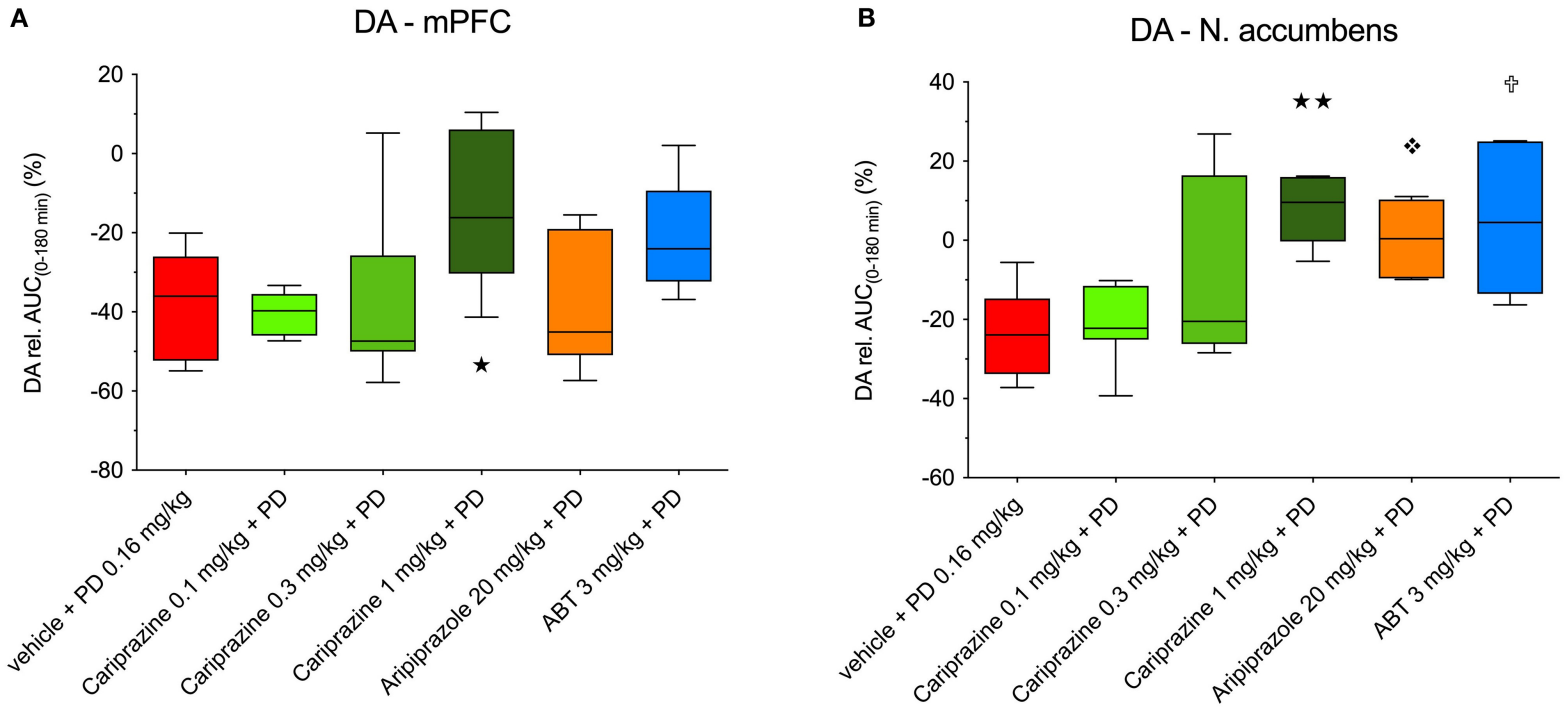
The overall propensity of cariprazine, aripiprazole and ABT-925 to counteract the (+)-PD 128907-induced decrease in basal extracellular levels of DA in the **(A)** mPFC and **(B)** nAcc shell of awake rats. (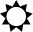

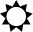
) *P* < 0.01, (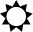) *P* < 0.05 for cariprazine (1 mg/kg) + PD; (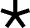) *P* < 0.05 for aripiprazole + PD; (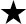) *P* < 0.05 for ABT-925 + PD; Kruskal-Wallis test followed by Dunn's multiple comparison test. ABT, ABT-925; PD, (+)-PD 128907.

Administration of (+)-PD 128907 in rats pretreated with vehicle decreased DA levels (Figures 4, 5A,B) in both regions. The most prominent effect was observed 40 min after (+)-PD 128907 injection, when the DA levels decreased to 57 and 45% in the mPFC and nAcc shell, respectively. In addition, the DA levels in the mPFC remained low until the end of the sampling period, whereas accumbal DA levels tended to return to pre-drug levels. The slight increases in DA levels in fractions collected at 0 and 20 min (compared to the basal samples collected at −60 to −20 min) were most likely caused by the handling stress and administration of drugs at these time intervals (arrows) as also confirmed by recordings of locomotor activity (see Supplementary Material).

Administration of cariprazine 20 min before (+)-PD 128907 administration caused a dose-dependent attenuation of (+)-PD 128907-induced decreases in DA levels in the mPFC (Figure 4A) and nAcc shell (Figure 4B).

While the lowest dose of 0.1 mg/kg had no effect, the intermediate dose of 0.3 mg/kg tended to attenuate the DA decrease in the mPFC at 40 min (*P* < 0.05) and a had minor effect in the nAcc shell at 180 min (*P* < 0.01). The highest dose of 1 mg/kg significantly (*P* < 0.01) prevented the (+)-PD 128907-induced DA decrease in the mPFC and completely abolished (*P* < 0.001) the effect of (+)-PD 128907 in the nAcc shell during the first 40-60 minutes and the end of the sampling period (140–180 min). In the mPFC, there was no significant difference between the groups for the treatment [F_(3, 24)_ = 2.275; P = 0.1056], but the interaction of time and treatment was significant [F_(36, 288)_ = 2.914; *P* < 0.001]. In the nAcc shell, there was a significant difference between the groups for both the treatment [F_(3, 24)_ = 6.082; *P* < 0.01] and the interaction of time and treatment [F_(36, 288)_ = 3.743; *P* < 0.001].

In the mPFC, ABT-925 tended to diminish the effect of (+)-PD 128907, particularly in the later stages of recording. However, the effect was only significant (*P* < 0.05) at 80 and 160 min post (+)-PD 128907 injection. Aripiprazole had no significant effects (Figure 4C). In the mPFC, there was no significant difference between the groups for the treatment [F_(2.18)_ = 2.648; P= 0.0981] or the interaction between time and treatment [F_(264, 216)_= 1.059; *P* < 0.3929]. In the nAcc shell, there was a significant difference between groups for both treatments [F_(2.18)_ = 13.69] but not for the interaction between time and treatment [F_(24, 216)_ = 1.183; P = 0.259].

The overall effects of pretreatment with cariprazine, aripiprazole, and ABT-925 on (+)-PD 128907-induced changes in the extracellular DA levels in the mPFC and nAcc shell are shown in Figures 5A,B, respectively.

The decreased DA levels were expressed as the relative area under the curve (AUC_(0−180*min*)_) for each treated group subtracted from the theoretical AUC_(0−180min)_ value (900%), which corresponds to nine 20-min basal samples from each group. The DA D_3_-receptor-preferring agonist (+)-PD 128907 decreased DA levels by 37.3 and 23.4% in the mPFC and nAcc shell, respectively. In the mPFC (Figure 5A), only the highest dose of cariprazine significantly (*P* < 0.05) diminished the effect of (+)-PD 128907, whereas aripiprazole had no significant effect. ABT-925 trended toward reversing the effect of (+)-PD 128907 on DA levels, although this was not statistically significant. In the nAcc shell (Figure 5B), all three compounds effectively counteracted the effect of (+)-PD 128907 on DA, with the highest dose of cariprazine having the most potent effect (*P* < 0.001), followed by ABT-925 (*P* < 0.01) and aripiprazole (*P* < 0.05).

#### Effects of (+)-PD 128907 and the Pretreatment With Cariprazine, Aripiprazole and ABT-925 on Levels of DOPAC and HVA in the mPFC and nAcc Shell of Awake Rats

Administration of (+)-PD 128907 in rats pretreated with vehicle did not induce long-lasting effects on DOPAC or HVA levels in either the mPFC or nAcc. As with DA, the most prominent effects were observed in the nAcc shell at 40 min after (+)-PD 128907 administration, with a 28% decrease for DOPAC and a 15% decrease for HVA. DOPAC and HVA returned to the basal levels within the following 60 min (data not shown).

**Figure 6:**
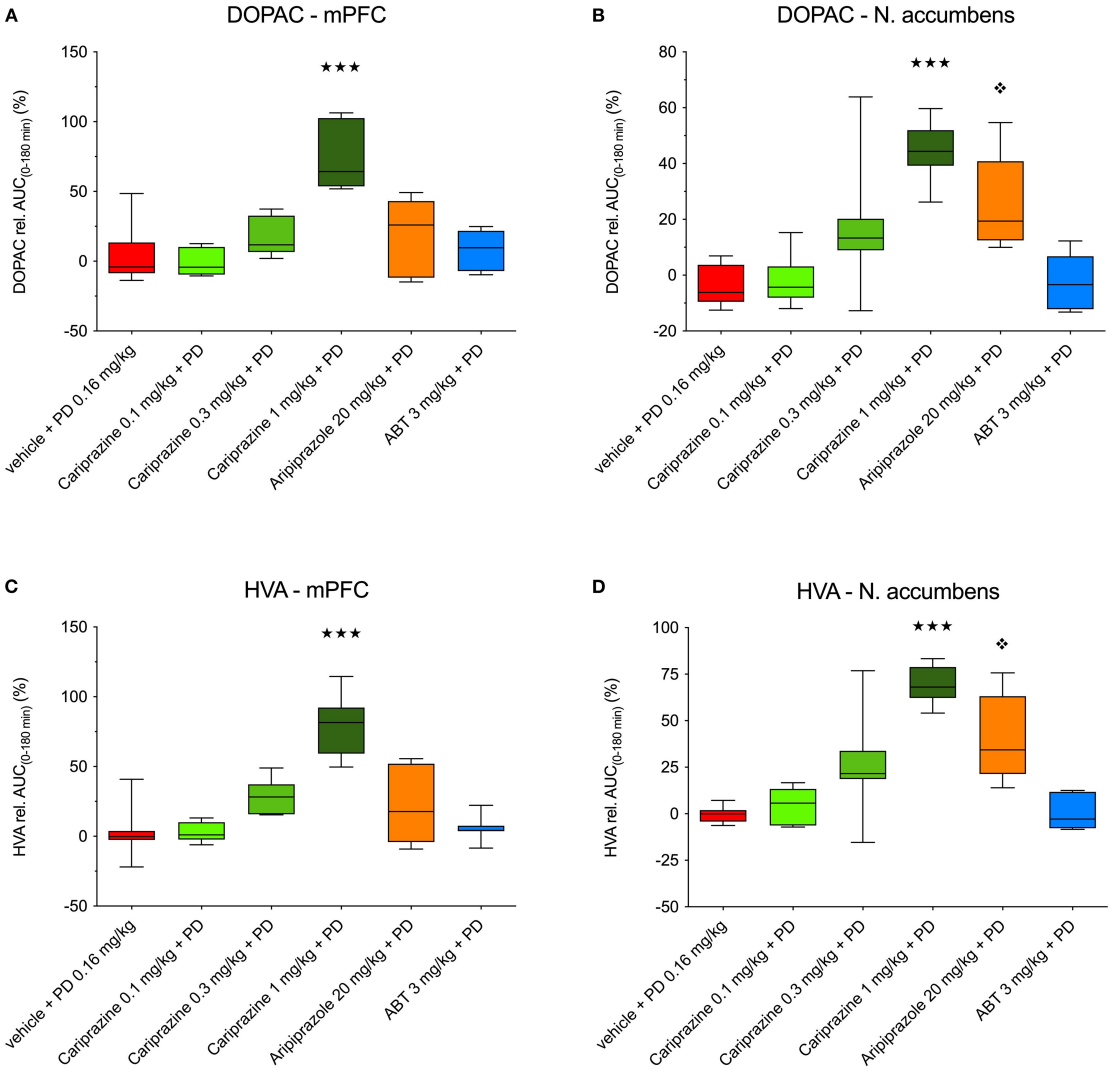
The overall effects of (+)-PD 128907 and the combined treatment with (+)-PD 128907 and cariprazine, aripiprazole or ABT-925 on DOPAC **(A,B)** and HVA **(C,D)** levels in the mPFC and nAcc shell of the awake rats. (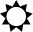

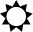

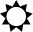
) *P* < 0.001, for cariprazine (1 mg/kg) + PD; (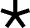) *P* < 0.05 for aripiprazole + PD; Kruskal–Wallis test followed by Dunn's multiple comparison test. ABT, ABT-925; PD, (+)-PD 128907.

The overall effects of pretreatment with cariprazine, ABT-925, and aripiprazole in combination with (+)-PD 128907 on DOPAC and HVA levels in the mPFC and nAcc shell are shown in Figure 6.

In the mPFC, the relative AUC_(0−180*min*)_ values for DOPAC and HVA were significantly (*P* < 0.001) different for the highest dose of cariprazine relative to (+)-PD 128907 alone (Figures 6A,C). In the nAcc shell, significant increases in the AUC_(0−180min)_ values for DOPAC and HVA were observed for two higher doses of cariprazine (*P* < 0.05 for 0.3 mg/kg, *P* < 0.001 for 1 mg/kg), as well as for aripiprazole (*P* < 0.01 for DOPAC; *P* < 0.001 for HVA) relative to (+)-PD 128907 alone (Figures 6B,D).

#### Effects of (+)-PD 128907 and Pretreatment With Cariprazine, Aripiprazole, and ABT-925 on Locomotor Activity of Rats Undergoing Microdialysis Sampling

The locomotor activity of rats was recorded simultaneously with microdialysis sampling in order to assess the effects of handling during drug administration and to identify other sources of stress potentially affecting the microdialysis data. The effects of cariprazine, aripiprazole, or ABT-925 pretreatment in combination with (+)-PD 128907 on the locomotor activity of rats undergoing microdialysis are shown in the supplementary data (Supplementary Figure S1A for cariprazine, Supplementary Figure S1B for aripiprazole and ABT-925). Following administration of the test compounds followed by (+)-PD 128907, there was an initial increase in motor activity during the first 20-min period, followed by a slowing of motor activity until it reached similar levels as shown by habituated rats at the start of the microdialysis sampling.

The forward movement of rats in their home cages was recorded in 5-min bins. Temporal increases in locomotor activity were observed between −20 to 0 min and 0 to 20 min. The differences between the drug-treated groups compared to the (+)-PD 128907 group during these periods are most likely caused by the stress induced by handling and administration of the test compounds per orally followed by subcutaneous (+)-PD 128907.

## Discussion

Dysregulated dopamine signaling is central to many models describing the pathophysiology of schizophrenia (36). In line with the corticolimbic distribution of D_3_ receptors, their role has long been studied in brain areas modulating cognitive functions or emotions. Interestingly, the extracellular DA level is twice as high in the nucleus accumbens of mice lacking the D_3_ receptor than in wild type animals (37), and pharmacological blockade of D_3_ receptors by selective antagonists increases extracellular levels of DA in the prefrontal cortex (38), a major regulator of the midbrain dopamine system. Distribution of the dopamine D_3_ receptors is mainly restricted to the limbic areas (Islands of Calleja, nucleus accumbens and ventral part of caudate nucleus) of the human and rodent brain, though low level of D_3_ receptor expression has also been described for cortical regions, particularly in the frontal cortex (20, 39–42). Here we report the first study of on dopamine D_3_ receptor associated behavior (i.e., huddling) with concurrent monitoring of extracellular dopamine in the mPFC and nAcc by dual microdialysis in freely moving rats.

In the huddling study, the D_3_ receptor-preferring agonist (+)-PD 128907 induced a marked (−72%) disruption of huddling behavior in the first 10 min after administration. Even the lowest dose of cariprazine (0.1 mg/kg) was more potent than aripiprazole at counteracting the disruption of huddling induced by (+)-PD 128907. Disruption of huddling behavior was almost completely abolished at a cariprazine dose of 0.3 mg/kg. Likewise, ABT-925, and to a lesser extent aripiprazole, were effective in blocking the (+)-PD 128907-induced effect. Pretreatment with cariprazine, ABT-925, and aripiprazole in the absence of (+)-PD 128907 had no effects on huddling duration compared to the vehicle-treated group. Because huddling behavior is believed to be mediated via DA D_3_ receptors (28–30), these behavioral results provide further evidence for cariprazine's preferential action at DA D_3_ receptors over D_2_ receptors *in vivo*.

Microdialysis sampling showed that cariprazine antagonized the effects of (+)-PD 128907 on basal extracellular levels of DA in both the mPFC and nAcc shell of awake rats. Pretreatment with cariprazine at the highest dose prevented the (+)-PD 128907-induced decrease in DA levels in the mPFC at various time points, as did ABT-925. Aripiprazole had no significant effect at any time point tested in this brain region. In the nAcc shell, all three compounds significantly reduced the (+)-PD 128907-induced decrease in DA levels. Levels of the DA metabolites DOPAC and HVA were not affected by (+)-PD 128907; however, levels of DOPAC and HVA were markedly elevated in the mPFC of rats treated with cariprazine (1 mg/kg) in (+)-PD 128907-treated rats. In the nAcc shell, DOPAC and HVA levels were also increased in rats treated with the lower 0.3 mg/kg dose of cariprazine, as well as in rats treated with aripiprazole in (+)-PD 128907-treated groups. These data suggest that D_3_ receptors may have a predominant role in regulating DA neurotransmission in the mPFC relative to D_2_ receptors, with D_3_ preferring agents having a greater impact in this region as compared to D_2_ receptor-preferring compounds. On the other hand, in the nAcc D_2_ and D_3_ receptors could both be involved as D_3_ receptor-preferring as well as D_2_ receptor-preferring agents affected DA neurotransmission.

The different involvement of dopamine D_2_ and D_3_ receptors in mediating neuronal responses has been suggested for the mPFC based on behavioral, as well as electrophysiology findings. Hodge et al. investigated the role of dopamine receptors in a reinforced response paradigm upon direct bilateral mPFC drug injection and found that the D_2_ antagonist raclopride did not modify the delayed response onset when co-administered with the D_2/3_ agonist quinpirole (43). Consistently, direct bilateral infusion of the D_3_ antagonists S33084 and SB277011 into the frontal cortex dose-dependently reversed the deficit in recognition induced by a delay, while the D_2_ antagonist, L741,626 had no effect. Moreover, such social recognition improving action of S33084 was specific to cortex as its injection into the nucleus accumbens was ineffective (44). Further, a bilateral injection of S33084 into the PFC prefrontal cortex increased the social novelty discrimination and novel object recognition (NOR) in rats, whereas no such effect was seen after intranigral injection, whereas the injection of L741626, a preferential dopamine D_2_ antagonist into the PFC (but not striatum) caused impairment in the NOR (45). Also, a distinct subset of pyramidal cells expressing dopamine D_3_ receptors has been described recently for the mPFC that may indirectly contribute to the predominant D_3_ receptor mediated DA neurotransmission in this cortical area. Within these neurons, dopamine D_3_ receptors via low-voltage-activated Ca_V_3.2 calcium channels localized on the axon initial segment regulate the ability of glutamatergic cells to generate high-frequency action potential bursts. Since neither D_2_, nor other dopamine receptors apart from the D_3_ are involved in regulating the excitability of this mPFC neuronal population, the D_3_ receptors seem to have unique actions in the mPFC (42).

The time courses of the behavioral and neurochemical effects of cariprazine were somewhat disparate in this study. The reasons for this disparity remain elusive. Disruption of huddling by (+)-PD 128907 occurred only during the first 10–20 min of the observational period, which is in agreement with the results of Kagaya et al. (28), while the extracellular DA level lowering effect of (+)-PD 128907 reached its maximum between 20–40 min after administration, in agreement with the results of Pugsley et al. (21) who described the short acting duration of (+)-PD 128907 for midbrain dopaminergic cells. Systemic dosing with the D_3_ agonist compound robustly, but transiently, suppressed the firing and bursting activity of dopamine neurons in the ventral tegmental area and pars compacta region of the substantia nigra and the spontaneous firing of dopamine neurons is restored within 5–10 min to 70–80% of the baseline firing (46, 47). These electrophysiology results are in line with the time-course of the behavioral effect of the current study as well as the data of former studies describing the effect of (+)-PD 128907 in induction of yawning or disruption of huddling as the latter also take place within 20–30 min (26, 28).

Data on locomotor activity from freely moving animals during microdialysis were collected that might have provided some hints on this discrepancy. However, the temporal increases in locomotor activity observed from −20 to 0 min and from 0 to 20 min were most likely due to the stress induced by handling and test compound administration. These stressors may have masked the effect of (+)-PD 128907 on social behavior observed in habituated rats. Nevertheless, the neurochemical changes by (+)-PD 128907 were evident in the microdialysis experiments and provided a solid basis for studying the effects of dopamine D_3_ receptor partial agonist and antagonist compounds for restoring or even further increasing DA levels in the mPFC and nAcc, respectively.

## Conclusion

Taken together, these data provide further evidence for cariprazine's preferential action *via* DA D_3_ receptors over D_2_ receptors in the rat brain *in vivo*. Cariprazine showed greater efficacy and potency than did aripiprazole at restoring a D_3_ receptor-mediated behavior (huddling). Further evidence for cariprazine's DA D_3_ receptor mechanism was its ability to counteract the effects of the D_3_ receptor-preferring agonist (+)-PD 128907 on decreased DA levels in both the rat mPFC and nAcc areas (unlike aripiprazole, which was only effective in the nAcc). These findings suggest that under conditions of cortical dopaminergic hypofunctionality, cariprazine can reverse this deficit. Cariprazine may therefore offer therapeutic benefits against a broad range of symptoms in schizophrenia and related disorders associated with reduced cortical DA neurotransmission, including cognitive deficits and negative/depressive symptoms.

## Data Availability Statement

The raw data supporting the conclusions of this article will be made available by the authors, without undue reservation.

## Ethics Statement

All animal experiments and protocols were approved by the Regional Ethical Committee at the Stockholm County court, following the directives of the Swedish Animal Welfare Act 1988:534 and complying with the Directive 2010/ 63/ EU (Council of the European parliament) The Guide for the Care and Use of Laboratory Animals and the Principles of Laboratory Animal Care (NIH publication No. 85-23).

## Author Contributions

NA, BK, BF, and JK were involved in the study design, analysis, and interpretation of data, the decision to present the results, and contributed to writing the manuscript. JK, F-HW, FI, and SY were involved in all experiments. All authors contributed to the article and approved the submitted version.

## Funding

This work was supported by funding from Allergan (Madison, NJ, USA), and Gedeon Richter Plc (Budapest, Hungary).

## Conflict of Interest

JK, F-HW, FI, and SY are employees of Pronexus Analytical AB. NA is an employee of Allergan (AbbVie). BK and BF are employees of Gedeon Richter Plc.

## Publisher's Note

All claims expressed in this article are solely those of the authors and do not necessarily represent those of their affiliated organizations, or those of the publisher, the editors and the reviewers. Any product that may be evaluated in this article, or claim that may be made by its manufacturer, is not guaranteed or endorsed by the publisher.
