# Supplementary material for: Preferential Effects of Cariprazine on Counteracting the Disruption of Social Interaction and Decrease in Extracellular Dopamine Levels Induced by the Dopamine D3 Receptor Agonist, PD-128907 in Rats: Implications for the Treatment of Negative and Depressive Symptoms of Psychiatric Disorders
Source: Front Psychiatry. 2022 Jan 12;12:801641. doi: 10.3389/fpsyt.2021.801641 (PMC8789685; doi:10.3389/fpsyt.2021.801641)
Supplement: Supplementary file 1 [file Table_1.PDF]

	Vehicle + PD	Cariprazine 0.1 mg + PD	Cariprazine 0.3 mg + PD	Cariprazine 1.0 mg + PD	Aripiprazole 20 mg + PD	ABT-925 3 mg + PD
**DA** (fmol/10 µl)						
mPFC	4.866 ± 0.382	4.671 ± 0.876	4.093 ± 0.237	3.580 ± 0.271	4.071 ± 0.640	3.654 ± 0.447
nAcc	19.77 ± 2.783	20.41 ± 2.287	21.26 ± 5.852	24.55 ± 2.469	22.30 ± 3.016	20.83 ± 2.302
**DOPAC** (fmol/10 µl)						
mPFC	419 ± 65	291 ± 30	384 ± 135	327 ± 72	397 ± 99	349 ± 26
nAcc	6034 ± 1010	4261 ± 541	4910 ± 776	5930 ± 850	4856 ± 961	5037 ± 1109
**HVA** (fmol/10 µl)						
mPFC	877 ± 126	711 ± 91	693 ± 127	806 ± 183	786 ± 60	781 ± 76
nAcc	2466 ± 293	2121 ± 278	1889 ± 264	2376 ± 408	2071 ± 253	2083 ± 239

	Vehicle + PD	Cariprazine 0.1 mg + PD	Cariprazine 0.3 mg + PD	Cariprazine 1.0 mg + PD	Aripiprazole 20 mg + PD	ABT-925 3 mg + PD
**mPFC**						
DA	4.87 ± 0.38	4.67 ± 0.88	4.09 ± 0.24	3.58 ± 0.27	4.07 ± 0.64	3.65 ± 0.45
DOPAC	419 ± 65	291 ± 30	384 ± 135	327 ± 72	397 ± 99	349 ± 26
HVA	877 ± 126	711 ± 91	693 ± 127	806 ± 183	786 ± 60	781 ± 76
**nAcc**						
DA	19.77 ± 2.78	20.41 ± 2.29	21.26 ± 5.86	24.55 ± 2.47	22.30 ± 3.02	20.83 ± 2.30
DOPAC	6034 ± 1010	4261 ± 541	4910 ± 776	5930 ± 850	4856 ± 961	5037 ± 1109
HVA	2466 ± 293	2121 ± 278	1889 ± 264	2376 ± 408	2071 ± 253	2083 ± 239

Table S1.
The basal extracellular levels of DA, DOPAC, and HVA (expressed in fmol/10 µl) in the microdialysates from vehicle and drug-treated rats.
