# Supplementary material for: Preferential Effects of Cariprazine on Counteracting the Disruption of Social Interaction and Decrease in Extracellular Dopamine Levels Induced by the Dopamine D3 Receptor Agonist, PD-128907 in Rats: Implications for the Treatment of Negative and Depressive Symptoms of Psychiatric Disorders
Source: Front Psychiatry. 2022 Jan 12;12:801641. doi: 10.3389/fpsyt.2021.801641 (PMC8789685; doi:10.3389/fpsyt.2021.801641)
Supplement: Supplementary file 2 [file Image_1.PDF]

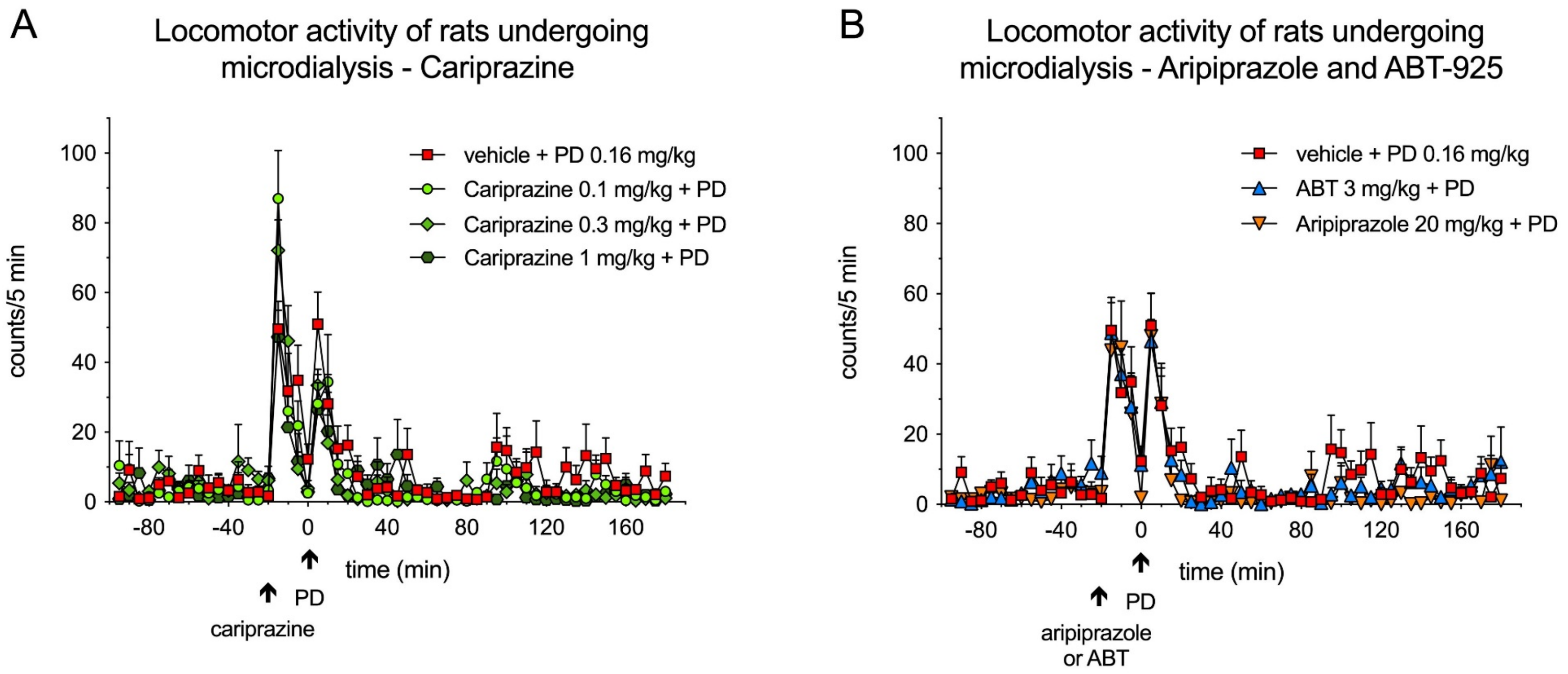

Figure S1.
The effects of A) cariprazine, and B) aripiprazole or ABT-925 pretreatment in combination with (+)-PD 128907 on the locomotor activity of rats undergoing microdialysis. There were no significant differences between the (+)-PD 128907-treated group and the (+)-PD 128907+drug-treated groups as revealed by two-way RP ANOVA followed by Bonferroni´s multiple comparison test.
